# Study on the physicochemical properties and antioxidant activity of hawthorn pectin extracted by ultrasound-assisted electrolysis of water

**DOI:** 10.1016/j.ultsonch.2026.108012

**Published:** 2026-08-19

**Authors:** Zishuai Liu, Jiamin Zhang, Congxuan Fei, Dandan Li, Hong Zhu, Jie Zhou, Guangxian Wang, Yafei Zheng, Xinming Liu, Jianhua Xiu

**Affiliations:** a College of Food and Biology, Hebei University of Science and Technology, Shijiazhuang, Hebei 050000, China; b Hebei Hawthorn Processing Technology Innovation Center, Chengde, Hebei 067300, China; c Hebei Max Biotechnology Co., Lt, Hengshui, Hebei 053900, China; d Hebei Chengde Shenli Food Co., Ltd, Chengde, Hebei 067400, China

**Keywords:** Hawthorn pectin, Electrolyzed water, Extraction, Physicochemical properties, Antioxidant activity

## Abstract

This study systematically compared ultrasound-assisted electrolyzed water extraction (UAEWE), ultrasound-assisted extraction (UAE), traditional acid extraction (AE), and commercially available hawthorn pectin (CHP) in terms of pectin yield, physicochemical properties, structural characteristics, and antioxidant activity. The objective was to evaluate the feasibility of UAEWE as a sustainable alternative for hawthorn pectin production and explore its potential applications. Results indicated that UAEWE-extracted pectin achieved the highest yield (7.21 ± 0.13%) and galacturonic acid (GalA) content (87.35 ± 0.37 g/100 g), significantly surpassing UAE (5.96 ± 0.06%; 82.28 ± 0.49 g/100 g) and AE (4.53 ± 0.15%; 76.08 ± 0.96 g/100 g). However, UAEWE-extracted pectin exhibited lower esterification degree (63.13 ± 0.08%), weight-average molecular weight (223.92 ± 4.22 kDa), and particle size (3.56 ± 0.45 μm) than the other samples. Monosaccharide composition analysis revealed similar sugar profiles among all samples, although relative proportions differed. Microstructural characterization showed that UAEWE-extracted pectin possessed a highly porous architecture, consistent with its superior extraction yield. FTIR and NMR analyses confirmed no significant structural alterations among the pectin samples. Rheological measurements demonstrated that UAEWE-extracted pectin exhibited the lowest apparent viscosity at 1% concentration, pH 3, and low shear rates. Moreover, UAEWE-derived pectin showed enhanced antioxidant activity compared with UAE, AE, and CHP samples. These findings highlight UAEWE as a promising extraction strategy for hawthorn pectin, offering reduced acid usage, cost-effectiveness, and environmental sustainability. The resulting pectin, characterized by high GalA content and antioxidant capacity, shows potential for bioactive compound encapsulation and functional food applications.

## 1 Introduction

Pectin, a class of polysaccharides located in the plant cell wall and the intercellular layer membrane, plays a pivotal role in the adhesion of adjacent cells [1]. Pectin is a structurally complex acidic polysaccharide composed mainly of three major structural domains: homogalacturonan (HG), rhamnogalacturonan I (RG-I), and rhamnogalacturonan II (RG-II) [2]. The HG region consists of linear chains of galacturonic acid (GalA) linked by α-1,4 glycosidic bonds, and its methyl esterification degree is closely associated with pectin gelation [2]. Specifically, high-methoxyl pectins (DE > 50%) form gels through hydrogen bonding and hydrophobic interactions under acidic conditions with high soluble solids content, whereas low-methoxyl pectins (DE < 50%) form calcium-mediated gels. The RG-I region features a backbone of repeating disaccharide units [→2)-α-L-Rha-(1 → 4)-α-D-GalA-(1 → ] with arabinan- and galactan-rich side chains attached to the rhamnose residues [3]. These neutral sugar side chains are known to contribute to the solubility, viscosity, emulsifying properties, and biological activities of pectin, such as immunomodulatory and antioxidant effects. The RG-II region, although present in a smaller proportion, is a structurally complex domain containing rare sugars such as apiose, aceric acid, and KDO (3-deoxy-D-*manno*-oct-2-ulosonic acid), and is important for maintaining structural stability and molecular conformation through its ability to form borate diester cross-links [4]. Recent studies have also shown that ultrasound-assisted extraction can modify the molecular characteristics, rheological behavior, and functional properties of pectins from different plant sources [1], [2]. Therefore, variations in these structural domains may partly explain the differences in the physicochemical and functional properties of extracted pectins. Natural pectin is primarily composed of three structural domains [5]. Among numerous natural plant resources, pectin has emerged as one of the most promising components owing to its unique structure and extensive application value. As previously stated, pectin is a structurally complex polysaccharide mainly sourced from plants such as citrus, grapefruit, and apple. Hawthorn occupies a distinct position in the rich and diverse plant kingdom. Hawthorn, belonging to the Rosaceae family, is widely distributed in China, North America, and Europe. It is rich in various components, including hawthorn acid, flavonoids, polysaccharides, proteins, minerals, and diverse vitamins [6]. In particular, hawthorn has a pectin content of up to 6.4%, suggesting that hawthorn may serve as a potential source for pectin recovery and utilization, particularly as an alternative to conventional sources [7]. This hawthorn pectin also encompasses diverse biological activities, including antioxidant [8], anti-saccharification [9], anti-tumor [10], and lipid-lowering [11]. With the increasing application of pectin in food, medicine, and other fields, the demand in the pectin market is growing annually.

To fulfill this demand and tackle the issue of inadequate supply, it is of great urgency to develop efficient pectin extraction methods. Even though traditional hawthorn pectin extraction techniques such as hot water extraction and acid extraction are widely utilized, they are plagued by drawbacks such as extended processing durations and high energy consumption. Consequently, researchers have delved into a variety of emerging green extraction technologies, including ultrasound-assisted extraction [12], enzymatic extraction [13], microwave-assisted extraction [14] and electrolyzed water extraction. At present, there are few literature reports on the extraction of pectin by electrolyzed water. Wang et al. (2020) took pomegranate peel residues as the research object and employed the electrolyzed water method to extract pectin from pomegranate peel residues, and then analyzed its substance composition and structural characteristics [15]. By comparing it with the extraction using ultrapure water, the extraction yield increased by 2.2%. Moreover, the pectin obtained by the electrolyzed water extraction method had a lower molecular mass and a higher mass fraction of D-galacturonic acid. Electrolytic water is a potential antibacterial agent with strong bactericidal effects, posing a threat to the membrane function of *S. aureus* and *Escherichia coli* [16]. At the same time, the active factors in electrolyzed water may also destroy the proteins in the cell wall, promote the dissolution of hawthorn pectin, and increase hawthorn pectin production. Compared with traditional chemical reagents, electrolyzed water has the advantages of low cost, safety, environmental protection, health, and non-toxicity [17]. Therefore, the use of electrolytic water instead of other strong acid reagents as the extraction solvent of hawthorn pectin is consistent with the demand for greener extraction processes. Recent studies have shown that green extraction systems, including ultrasound-assisted extraction and alternative solvent-based extraction, can improve pectin recovery and regulate the structural and functional properties of pectin from plant sources [1], [18], [19], [20], [21].

Despite these advances, the application of electrolyzed water as a green extraction medium for hawthorn pectin remains largely unexplored. In particular, systematic investigations on how electrolyzed water extraction, especially when combined with ultrasound assistance, affects the physicochemical, structural, rheological, and antioxidant properties of hawthorn pectin are still lacking. To address this gap, the present study was designed to develop and evaluate an ultrasound-assisted electrolyzed water extraction (UAEWE) method for recovering pectin from hawthorn. The specific objectives of this work were to: (i) compare the extraction efficiency and basic chemical characteristics of pectins obtained by UAEWE, UAE, AE, and CHP; (ii) characterize the structural features of the extracted pectins, including degree of esterification (DE), molecular weight (Mw), monosaccharide composition, Fourier transform infrared (FTIR) spectra, and nuclear magnetic resonance (NMR) profiles; and (iii) evaluate and correlate the rheological behavior and antioxidant activity of the pectin samples with their structural properties. By systematically investigating these aspects, this study aims to provide a comprehensive understanding of the potential of UAEWE as a sustainable and efficient alternative for hawthorn pectin production and to explore its possible applications in the food industry.

## 2 Materials and methods

### 2.1 Chemicals and materials

Dried hawthorn fruits were purchased from Shenwei Pharmaceutical Co., Ltd. (Shijiazhuang, China). The dried hawthorn fruits were ground using a high-speed pulverizer, passed through an 80-mesh sieve, and stored in sealed bags until further use. All reference standards with a purity exceeding 99.8% were purchased from Yuanye Biotechnology Co., Ltd., China. Other analytical-grade chemicals were purchased from Tianjin Damao Chemical Reagent Co., Ltd. (Tianjin, China).

### 2.2 Extraction methods of hawthorn pectin

**Fig. 1:**
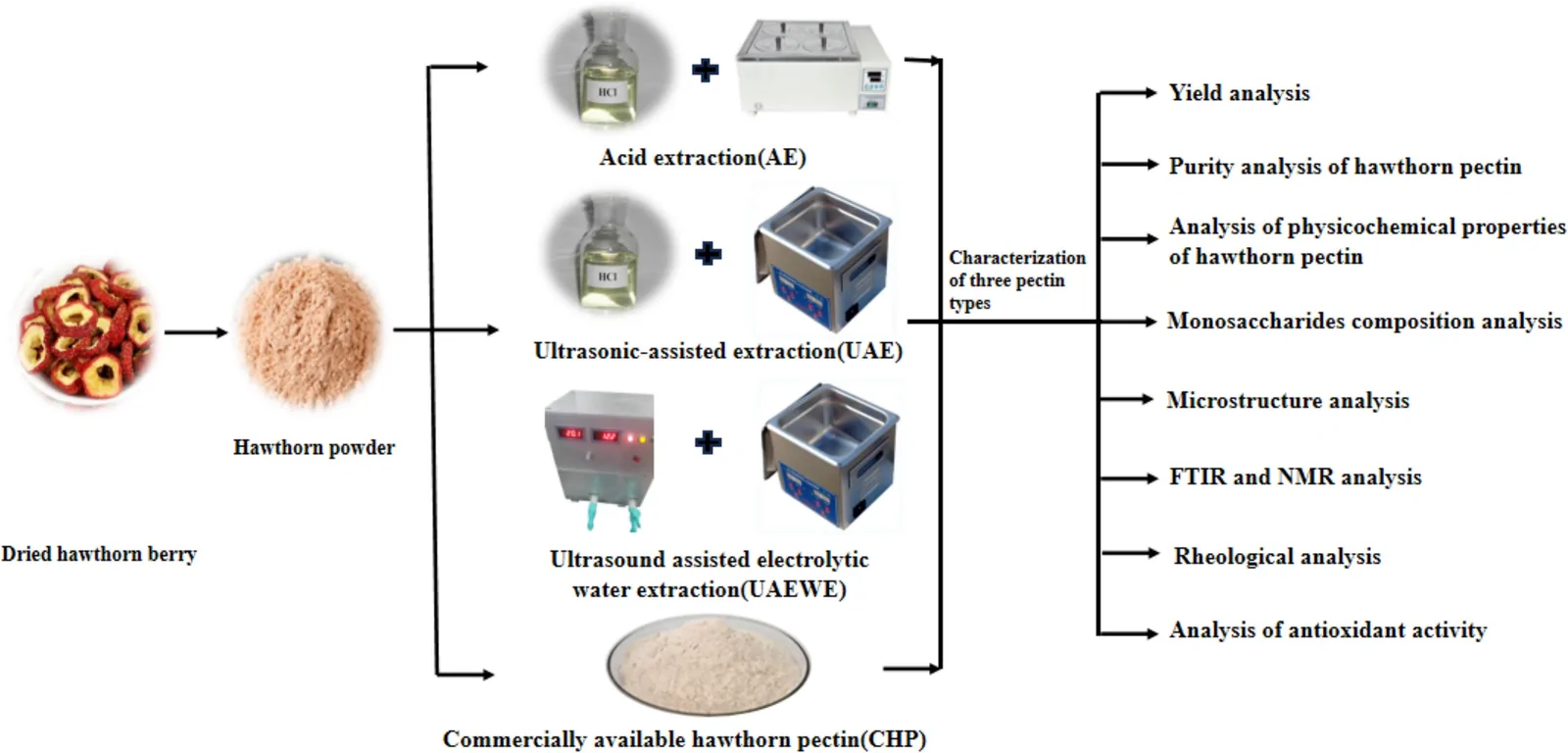
Experimental flow chart of hawthorn pectin extraction and characterization.

The overall experimental design and extraction workflow are presented in Fig. 1**.** Briefly, hawthorn pectin was prepared using three extraction methods: ultrasound-assisted electrolyzed water extraction (UAEWE), ultrasound-assisted extraction (UAE), and conventional acid extraction (AE). Commercial hawthorn pectin (CHP) was included as a reference sample. The method-specific extraction conditions are described in 2.2.1, 2.2.2, 2.2.3, while all extracts were subsequently purified using the common procedure described in Section 2.2.4.

#### 2.2.1 Ultrasound-assisted electrolytic water extraction (UAEWE)

Electrolyzed water preparation: 13.0 g of sodium chloride was dissolved in 12 L of deionized water and transferred into the feed tube. The electrolyzed water generator (Model XY-L5-1000, Baoji Xinyuguang Electromechanical Co., Ltd., Shijiazhuang, China) was then activated with a current setting of 20 A and voltage of 20 V. After electrolysis for 15 min, electrolyzed water with a pH of approximately 1.5 was obtained.

Hawthorn powder (2.0 g) and electrolyzed water (60 mL, pH 1.5) were added to a 150 mL conical flask. Ultrasound-assisted extraction was then performed at 80 °C for 60 min with an ultrasonic power of 100 W.

#### 2.2.2 Ultrasound-assisted extraction (UAE)

The UAE extraction procedure was performed according to the method of Senit et al. [22], with slight modifications. For UAE extraction, hawthorn powder (2.0 g) was dispersed in 60 mL of deionized water in a 150 mL conical flask. The pH of the suspension was adjusted to 1.5 using 1.5 mol/L hydrochloric acid solution. Ultrasound-assisted extraction was then conducted at 80 °C for 60 min with an ultrasonic power of 100 W.

#### 2.2.3 Acid extraction (AE)

The AE extraction procedure was performed according to the method described by Yu et al. [23], with slight modifications. For AE extraction, hawthorn powder (2.0 g) was dispersed in 60 mL of deionized water adjusted to pH 1.5 in a 150 mL conical flask. The suspension was extracted in a water bath at 80 °C for 60 min.2.2.4. Post-extraction purification

Following extraction, the suspensions obtained by UAEWE, UAE, and AE were centrifuged at 4500 r/min for 15 min, and the supernatants were collected. Pectin was precipitated from the supernatants by the addition of 95% ethanol, followed by incubation at 4 °C overnight. The resulting precipitates were recovered by centrifugation at 5000 r/min for 10 min and redissolved in a small volume of distilled water. The pectin solutions were subsequently transferred into dialysis tubing with a molecular weight cutoff (MWCO) of 3.5 kDa and dialyzed against distilled water. Dialysis was performed for 24 h for the UAEWE and UAE samples and for 48 h for the AE sample, with the dialysis water replaced every 4 h. After dialysis, the purified pectin samples were freeze-dried for 24 h and stored until further analysis.

#### 2.2.4 Commercial hawthorn pectin (CHP)

The commercially available hawthorn pectin used as a reference sample was purchased from Zhongyuan Biotechnology Company.

### 2.3 Determination of galacturonic acid content and calculation of pectin yield

For the analyses described in this section and Section 2.5, a stock pectin solution was prepared as follows: accurately weighed dried pectin sample (approximately 10 mg) was dissolved in deionized water and diluted to a final volume of 100 mL in a volumetric flask.

#### 2.3.1 Determination of galacturonic acid content

The galacturonic acid (GalA) content of hawthorn pectin was determined using the carbazole colorimetric method with D-galacturonic acid as the standard, according to the method of Galambos [24], with slight modifications. D-Galacturonic acid standard solutions at concentrations of 10, 20, 40, 60, 80, and 100 μg/mL were prepared. To 1 mL of each standard solution, 2 mL of 0.025 M sodium tetraborate in concentrated sulfuric acid was added, and the mixture was heated in a boiling water bath for 10 min. After cooling to room temperature, 50 μL of 0.15% carbazole reagent in absolute ethanol was added, and the mixture was heated again in a boiling water bath for 10 min. The absorbance was measured at 530 nm. The calibration curve showed good linearity within the concentration range of 10–100 μg/mL, with the equation *Y* = 0.0105*x* + 0.008 (*R^2^* = 0.99930). The GalA content of hawthorn pectin was calculated according to Equation (1):

(1) $$\mathrm{GalAcontent}\left(g/100g\right)=\frac{\mathrm{CVX}}{m\times 10^{6}}\times 100$$

where *C* represents the GalA-equivalent concentration of the tested pectin solution (mg/L), *V* represents the final volume of the tested solution (mL), *X* represents the dilution factor, and *m* represents the dry mass of the pectin sample used for analysis (g).

#### 2.3.2 Calculation of pectin yield

The pectin yield, defined as the percentage of galacturonic acid (GalA) equivalents recovered from the initial dry hawthorn powder, was calculated using the GalA-equivalent concentration obtained as described in Section 2.3.1, according to Equation (2):

(2) $$G\left(\%\right)=\frac{\mathrm{CVX}}{M\times 10^{6}}\times 100$$

where *C* represents the GalA-equivalent concentration of the tested pectin solution (mg/L), *V* represents the final volume of the tested solution (mL), *X* represents the dilution factor, and *M* represents the mass of dry hawthorn powder used for extraction (g).

### 2.4 Full‑wavelength UV scanning of pectin

Deionized water was used to prepare a 0.02% pectin solution. Then, a UV spectrophotometer was employed to conduct full-wavelength scanning within the wavelength range from 200 to 800 nm with a step size of 0.2 nm.

### 2.5 Determination of degree of esterification

The degree of esterification (DE) of the pectin samples was determined using a previously reported titration method [25], with slight modifications. An aliquot of the stock pectin solution (prepared as described in Section 2.3) was used directly for the determination. Briefly, 100 mL of the pectin solution was transferred to a beaker. Phenolphthalein was used as the indicator, and the solution was titrated with standardized 0.1 mol/L NaOH until a faint pink color appeared and persisted for 30 s. The volume of NaOH consumed was recorded as V_1_, corresponding to the free carboxyl groups.

Subsequently, 15 mL of 0.25 mol/L NaOH was added to the solution, and the mixture was stirred at room temperature for 30 min to saponify the esterified carboxyl groups. After saponification, 15 mL of 0.25 mol/L hydrochloric acid was added to neutralize the excess alkali. The resulting solution was then back-titrated with standardized 0.1 mol/L NaOH until the faint pink color persisted for 30 s. The volume of NaOH consumed in the second titration was recorded as V_2_, corresponding to the esterified carboxyl groups. Finally, the DE was calculated using the following Equation (3):

*DE (%)* = $\frac{V_{2}}{V_{1}+V_{2}}$× 100 (3).

where *V_1_* represents the volume of NaOH consumed for titrating free carboxyl groups, and *V_2_* represents the volume of NaOH consumed after saponification of esterified carboxyl groups.

### 2.6 Determination of molecular weight distribution of hawthorn pectin

The molecular weight of hawthorn pectin was determined by high-performance size-exclusion chromatography (HPSEC) according to the method of Lv et al. (2024) [26], with slight modifications. A calibration curve was first established using dextran standards with different molecular weights (1,000, 2,000, 5,000, 10,000, 40,000, and 100,000 Da). Each dextran standard was dissolved in 0.05 M NaCl solution at a concentration of 1 mg/mL and injected under the same chromatographic conditions as described below. The calibration curve was constructed by plotting the logarithm of the molecular weight (log Mw) against the retention time. The fitted equation was *Y* =  − 0.2225*x* + 11.457 (*R^2^* = 0.9945), where *Y* represents log Mw and *x* represents the retention time (min).

For sample analysis, 5 mg of dried pectin sample was accurately weighed and dissolved in 1 mL of 0.05 M NaCl solution to prepare a pectin solution at a concentration of 5 mg/mL. The solution was ultrasonicated at 100 W for 10 min to ensure complete dissolution, and then centrifuged at 12,000 r/min for 10 min to remove any insoluble residues. The supernatant was carefully collected and filtered through a 0.22 μm membrane filter prior to chromatographic analysis.

Chromatographic analysis was performed using a Shimadzu LC-10A high-performance liquid chromatography system equipped with BRT105-103–101 serial gel columns (8 × 300 mm) and a refractive index (RI) detector. The mobile phase was 0.05 M NaCl solution, which was filtered through a 0.45 μm membrane and degassed before use. The flow rate was set at 0.8 mL/min, and the column temperature was maintained at 40 °C. The injection volume was 25 μL, and the run time was 60 min for each sample.

The weight-average molecular weight (Mw), number-average molecular weight (Mn), and polydispersity index (Mw/Mn) were calculated from the chromatographic data using the calibration curve established with dextran standards. All samples were analyzed in triplicate, and the results were expressed as mean ± standard deviation.

### 2.7 Determination of particle size distribution of hawthorn pectin

The particle size distribution of hawthorn pectin was determined according to the method of Chen et al. (2019) [6], with slight modifications.

Sample preparation: Dried pectin sample (200 mg) was accurately weighed and transferred into a 100 mL volumetric flask. The sample was dissolved and diluted to volume with deionized water to obtain a pectin solution at a final concentration of 2 mg/mL. The solution was gently stirred at room temperature for 2 h to ensure complete dissolution and to obtain a homogeneous dispersion prior to measurement.

Measurement conditions: The particle size distribution was measured using a nanoparticle size analyzer (SZ-100 V2, HORIBA, Japan) equipped with a 633 nm He–Ne laser. Measurements were performed at a fixed scattering angle of 90° and a controlled temperature of 25 ± 0.1 °C. Each sample was equilibrated for 120 s before measurement to ensure temperature stability. Each sample was measured in triplicate, with 10 runs per measurement.

Data reporting: The results were expressed as the intensity-weighted mean hydrodynamic diameter (Z-average) and polydispersity index (PDI). All data are presented as mean ± standard deviation (n = 3).

### 2.8 Monosaccharide composition

The monosaccharide composition of hawthorn pectin was analyzed by high-performance liquid chromatography (LC-20A, Shimadzu Corporation, Kyoto, Japan) equipped with a UV detector, according to the method of Li et al. [27], with slight modifications. The samples were first hydrolyzed using 2 M trifluoroacetic acid at 110 °C for 4 h. After hydrolysis, the hydrolysates were derivatized with 0.5 M 1-phenyl-3-methyl-5-pyrazolone (PMP) and 0.3 M NaOH at 70 °C for 30 min. Excess PMP was removed by chloroform extraction. Chromatographic separation was carried out on a ZORBAX SB-C18 reversed-phase column (250 mm × 4.6 mm, 5 μm, Agilent, USA). The mobile phase was composed of 20 mM KH_2_PO_4_ solution (A) (pH was adjusted to 6, prepared using ultrapure water) and acetonitrile (B) (ACN). Isocratic elution was performed with mobile phases A and B at a ratio of 80:20 (v/v), and the run time was set to 50 min. The flow rate, column temperature, and injection volume were set at 0.8 mL/min, 40 °C, and 20 μL, respectively. Based on the monosaccharide composition, the relative proportions of homogalacturonan (HG) and rhamnogalacturonan-I (RG-I) regions were estimated [28].

HG (%) *=*$\;\frac{\mathrm{GalA}/194-Rha/164}{\mathrm{GalA}/194}$ × 100 (4).

RG-I (%) *=* $\frac{\mathrm{Rha}/164}{\mathrm{GalA}/194}$ × 100 (5).

where GalA and Rha represent the mass contents of galacturonic acid and rhamnose, respectively, and 194 and 164 are their corresponding molecular weights.

### 2.9 Scanning electron microscopy (SEM)

The morphology of hawthorn pectin samples was assessed through scanning electron microscopy (FE-SEM, MIRA3, Tescan). A suitable amount of dried hawthorn pectin samples was attached to the sample stage and coated with a conductive gold film. The accelerated voltage was set at 15 kV. Subsequently, a representative field of view was chosen for photographic documentation [29].

### 2.10 FTIR analysis

Freeze-dried samples of hawthorn pectin, which were extracted by different methods, were blended with KBr at a ratio of 1:100. After that, the mixture was thoroughly ground until it became homogeneous. Then, the uniformly ground mixture was evenly spread on a glass slide. Subsequently, the mixture was pressed to form a transparent pellet without any cracks. Following this, the sample was placed in the holder of the FTIR spectrometer (Jasco Inc., Easton, MO) for scanning. The scanning range was set from 750 to 4000 cm^−1^, the resolution was maintained at 4 cm^–1^, and the total number of scans was 32 [30].

The DE was calculated from the FTIR spectra using Equation (6) [31]:

DE (%) *=*$\;\frac{A_{1740}}{A_{1740}+A_{1630}}$ × 100 (6).

where A_1740_ and A_1630_ represent the peak areas at 1740 and 1630 cm^–1^ corresponding to esterified and free carboxyl groups, respectively.

### 2.11 Nuclear magnetic resonance (NMR) analysis

The NMR spectra were obtained with a Bruker 600 MHz AVANCE III NMR spectrometer (Bruker, Rheinstetten, Germany). Each dry pectin sample was dissolved in D_2_O and scanned 16 times at 25 ℃. The equilibrium concentration of the pectin solution in D_2_O was set to 10 mg/mL [29].

### 2.12 Measurement of rheological properties of pectin

The preparation of a pectin dispersion involved dissolving the pectin specimen in distilled water, resulting in a solution with a mass concentration of 1% (w/w). Subsequently, the mixture was refrigerated at 4 °C and remained there throughout the night. In the shear rheology experiment, a parallel plate structure (diameter d = 50 mm, gap size 1.0 mm) was loaded into a strain-controlled rheometer (HAAKE MARS 40 rheometer, USA). Before each measurement, the sample was pre-sheared at a constant shear rate of 1 s^–1^ for 60 s to eliminate loading history effects and establish a consistent initial state. After this pre-shear step, the specimen was allowed to rest for an additional 60 s to enable stress relaxation before the viscosity measurement was initiated. Viscosity measurements were then performed over a shear rate range of 0.001 to 100 s^−1^ during the upward flow scanning phase. The relationship between shear stress and shear rate was fitted using the Power-law model according to the method of Méndez et al. [32], and the rheological parameters were calculated according to Equation (7):

*σ* = *Kγ̇*^n^ (7).

where *σ* is the shear stress (Pa), *K* is the consistency coefficient (Pa·s^n^), *γ̇* is the shear rate (s^–1^), and n is the flow behavior index. A value of *n* < 1 indicates shear-thinning behavior, *n* = 1 represents Newtonian behavior, and *n* > 1 indicates shear-thickening behavior.

### 2.13 Analysis of the antioxidant activity of pectin

#### 2.13.1 DPPH assay

Referring to the method of Li et al. [33], the in vitro antioxidant activity of hawthorn pectin was determined with Vitamin C (VC) as a positive control. Accurately weighed 2,2-diphenyl-1-picrylhydrazyl (DPPH) and prepared a solution with anhydrous ethanol at a concentration of 0.2 mmol/L. Pectin sample solutions of different mass concentrations (2.5, 2.0, 1.5, 1.0, 0.5 mg/mL) were prepared and mixed with the same volume of DPPH. Pure water and ethanol were used as blank controls. The mixture was shaken thoroughly and incubated in the dark for 25 min. The absorbance was measured at 517 nm, and the DPPH radical scavenging activity was calculated according to Equation (8):

DPPH radical scavenging activity (%) = (1 *−*$\;\frac{A_{2}-A_{1}}{A_{0}}$) × 100 (8).

where A_0_ represents the absorbance of the pure-water control, A_1_ represents the absorbance of the ethanol blank, and A_2_ represents the absorbance of the sample.

#### 2.13.2 ABTS assay

Referring to the method of Li et al. [33], the in vitro antioxidant activity of hawthorn pectin was determined with VC as a positive control. Equal volumes of 2,2′-azino-bis (3-ethylbenzothiazoline-6-sulfonic acid) (ABTS) solution (7 mmol/L) and potassium persulfate solution (2.45 mmol/L) were mixed and kept overnight at 4 °C to generate ABTS^+^ radicals. The absorbance of the ABTS working solution was adjusted to 0.70 ± 0.02 at 734 nm using phosphate buffer (pH 7.4). Then, 1 mL of pectin sample solution at different concentrations (2.5, 2.0, 1.5, 1.0, and 0.5 mg/mL) was mixed with 3 mL of ABTS working solution. After thorough mixing, the reaction mixture was incubated in the dark for 5 min. The absorbance was measured at 734 nm, and the ABTS radical scavenging activity was calculated according to Equation (9):

ABTS radical scavenging activity (%) =(1-$\frac{A_{2}-A_{1}}{A_{0}}$)×100 (9).

where A_0_ represents the absorbance of the pure-water control, A_1_ represents the absorbance of the phosphate-buffer blank, and A_2_ represents the absorbance of the sample.

#### 2.13.3 Ferric reducing antioxidant power assay

The in vitro antioxidant activity of hawthorn pectin was determined using VC as a positive control, following a previously reported method [33]. For the ferric reducing power assay, 0.5 mL of pectin sample solution at different concentrations (2.5, 2.0, 1.5, 1.0, and 0.5 mg/mL) was mixed with equal volumes of phosphate buffer (pH 6.6) and potassium ferrocyanide solution (10 g/L). The mixture was incubated in a water bath at 50 °C for 25 min and then cooled to room temperature. Subsequently, 1.00 mL of trichloroacetic acid solution (100 g/L) was added and mixed thoroughly. After centrifugation, 1 mL of the supernatant was collected and mixed with 0.25 mL of ferric chloride solution (1 g/L). The reaction was carried out in the dark for 30 min, and the absorbance was measured at 700 nm. The reducing power of pectin was evaluated by comparing the absorbance of the sample with that of vitamin C.

### 2.14 Statistical analysis

All experimental results were replicated three times. The data are presented as mean ± standard deviation. The IBM SPSS 19.0 statistical software (SPSS, Inc., Chicago, IL, USA) was used to perform one-way analysis of variance (ANOVA) followed by Duncan's multiple range test. Differences were considered statistically significant at *p* < 0.05. Different lowercase letters in tables and figures indicate significant differences among samples at *p* < 0.05. Origin 2021 (Origin Lab Corp., MA, USA) was used for data plotting.

## 3 Results and discussion

### 3.1 UV analysis

UV–visible full‑wavelength scanning was performed on the three pectin samples (UAEWE, UAE, and AE) over the range of 200–800 nm. As shown in **Fig. S1** (Supplementary Material), none of the three pectin samples exhibited distinct absorption peaks at 260 nm or 280 nm, suggesting that the levels of UV‑absorbing impurities, such as nucleic acids and proteins, were relatively low in these pectin samples. However, it should be noted that UV spectroscopy alone cannot quantitatively confirm the complete absence of proteins or nucleic acids, as low concentrations of these impurities may not produce detectable absorption signals, and certain proteins lacking aromatic residues may not absorb at 280 nm. Similar UV spectral characteristics have been reported for ultrasound‑assisted pectin and polysaccharide samples, where weak or absent absorption in the 260–280 nm region was interpreted as indicative of low levels of UV‑absorbing impurities rather than as proof of absolute purity [21], [34]. The commercial sample CHP was not included in the UV analysis, as the primary purpose of this measurement was to compare the purification efficiency of the three extraction methods. Nevertheless, the GalA contents of all four pectin samples (including the commercial sample CHP) exceeded 65% (w/w) (Table 1), meeting the purity requirements for food‑grade pectin applications, which further supports the overall quality of the extracted pectins.

**Table 1:** Chemical and monosaccharide compositions of hawthorn-derived pectin.

	UAEWE	UAE	AE	CHP
Physicochemical indexes				
Yield (%)	7.21 ± 0.13^a^	5.96 ± 0.06^b^	4.53 ± 0.15^c^	—
DE by titration (%) DE by FTIR (%)	63.13 ± 0.08^c^ 60.32	67.47 ± 0.18^b^ 64.24	70.58 ± 0.23^a^ 66.75	65.58 ± 0.23^d^ 60.51
GalA content (g/100 g)	87.35 ± 0.37^a^	82.28 ± 0.49^b^	76.08 ± 0.96^c^	78.08 ± 0.96^c^
Mw (kDa)	223.92 ± 4.22^d^	263.04 ± 3.64^b^	290.91 ± 2.47^a^	235.24 ± 2.38^c^
Mn (kDa)	218.83 ± 3.46^d^	257.06 ± 3.87^b^	284.29 ± 1.98^a^	222.16 ± 2.45^c^
Mw/Mn	1.02 ± 0.01^a^	1.02 ± 0.02^a^	1.02 ± 0.01^a^	1.02 ± 0.03^a^
Particle size (μm) Monosaccharide composition	3.56 ± 0.45^d^	4.25 ± 0.32^c^	5.67 ± 0.21^a^	4.67 ± 0.21^b^
Rha (%)	3.03 ± 0.01^a^	2.97 ± 0.03^a^	3.16 ± 0.02^a^	2.16 ± 0.02^b^
GalA (%)	77.98 ± 0.10^a^	75.80 ± 0.12^b^	75.38 ± 0.16^c^	65.38 ± 0.16^d^
Gal (%)	2.86 ± 0.02^b^	2.67 ± 0.01^c^	3.04 ± 0.01^a^	2.04 ± 0.01^d^
Man (%)	0.11 ± 0.04^b^	0.13 ± 0.03^a^	0.06 ± 0.02^c^	0.07 ± 0.02^c^
Ara (%)	2.24 ± 0.06^b^	2.26 ± 0.02^b^	2.39 ± 0.04^a^	2.12 ± 0.04^a^
Glc (%)	18.79 ± 0.02^a^	16.17 ± 0.07^b^	15.96 ± 0.05^c^	13.42 ± 0.05^d^
HG (%)	95.30 ± 0.05^b^	95.36 ± 0.08^b^	95.05 ± 0.02^a^	96.09 ± 0.02^c^
RG-I (%)	4.69 ± 0.03^b^	4.63 ± 0.01^b^	4.94 ± 0.05^a^	3.91 ± 0.05^c^
Rha/GalA	0.04 ± 0.00^a^	0.04 ± 0.01^a^	0.04 ± 0.03^a^	0.03 ± 0.03^b^
(Ara + Gal)/Rha	1.68 ± 0.01^c^	1.65 ± 0.01^d^	1.72 ± 0.01^b^	1.92 ± 0.01^a^

DE values were determined by both titrimetric and FTIR methods. Titrimetric DE values are presented as mean ± SD (n = 3). FTIR DE values were calculated from the absorbance peak area ratio A_1740_/(A_1740_ + A_1630_) based on single FTIR measurement. Different superscript letters (a–d) in the same row indicate significant differences (*p* < 0.05). ‘—’ indicates that the yield was not determined for CHP because it was used as a commercial reference sample rather than a pectin sample extracted in this study. UAEWE: ultrasound-assisted electrolyzed water extraction; UAE: ultrasound-assisted extraction; AE: acid extraction; CHP: commercial hawthorn pectin.

### 3.2 The yield of hawthorn pectin

The yields of UAEWE, UAE, and AE were 7.21 ± 0.13%, 5.96 ± 0.06%, and 4.53 ± 0.15%, respectively, as shown in Table 1. There were significant differences in the yields among UAEWE, AE, and UAE (*p* < 0.05). The use of ultrasound-assisted electrolysis method to prepare pectin could markedly enhance the pectin yield. This was because the low pH value and high redox potential of acidic electrolyzed water could disrupt the cell matrix, resulting in increased membrane permeability. During electrolysis of water containing trace electrolytes (e.g., sodium chloride), the oxidation of water occurs at the anode to generate oxygen gas and hydrogen ions, while the reduction of water proceeds at the cathode to produce hydrogen gas and hydroxide ions. This intricate process culminates in the generation of acidic and alkaline electrolyzed water with distinct physicochemical properties. Specifically, the low pH of acidic electrolyzed water may facilitate the hydrolysis of ester bonds in pectin, thereby promoting its release from the plant cell matrix [15]. It was speculated that the active factors in electrolyzed water could also destroy proteins in the cell wall, while ultrasound further enhanced pectin dissolution and increased yield [35]. Compared with ultrasound-assisted acid extraction and traditional acid extraction methods, the use of acidic electrolyzed water could reduce the amount of hydrochloric acid, which also made outstanding contributions to reducing costs and green environmental protection.

The pectin yield obtained by UAEWE (7.21%) in this study was compared with previously reported values for hawthorn pectin under similar raw material conditions. Wu et al. (2018) reported that hydrochloric acid extraction of dried hawthorn powder yielded 3.07%–4.11% pectin depending on the cultivar [36]. Wang et al. (2023) optimized ultrasound-assisted extraction from hawthorn peel residue and obtained a yield of 6.15% under optimal conditions (74 °C, 425 W, 55 min) [37]. For comparison, Fu et al. (2024) reported that deep eutectic solvent extraction of hawthorn pectin achieved a yield of 4.33% [38], while recent studies on ultrasound-assisted ionic liquid extraction reached up to 7.93% [39]. However, such high yields are not directly comparable to those obtained from unprocessed dried hawthorn powder. Overall, the UAEWE yield (7.21%) is significantly higher than conventional acid extraction (3.07%-4.11%) and traditional UAE (6.15%) under similar raw material conditions, confirming the synergistic effect of acidic electrolyzed water and ultrasound in promoting pectin release from the plant cell matrix.

### 3.3 DE of hawthorn pectin

Table 1 shows that the degree of esterification of UAEWE is 63.13 ± 0.08%, that of UAE is 67.47 ± 0.18%, that of AE is 70.58 ± 0.23%, and that of CHP is 65.58 ± 0.16%. All four belong to the category of high-methoxyl pectin (HMP). This result was consistent with previous studies on hawthorn pectin. A previous study reported DE values of 54.16% and 53.34% for red hawthorn pectins extracted using acidic solvents [40]. Another study also showed that the extraction method could affect the DE of hawthorn pectin [26]. DE reflects the methyl and acetyl groups linked to galacturonic acid and is chiefly utilized for determining the gel properties of pectin [41]. The yields of hawthorn pectin were compared using three different processes, and the results showed that the esterification degree of UAEWE was lower than that of UAE, CHP, and that of AE was the highest. This might suggest that ultrasound has the potential to degrade polysaccharides, break glycosidic bonds, and result in a reduction in the esterification degree. Electrolytic water solvents can further degrade the methyl esterification groups in hawthorn pectin [42].

In addition to the titrimetric method, the DE of the pectin samples was also calculated from the FTIR peak area ratio (A_1740_/(A_1740_ + A_1630_)). The FTIR-derived DE values were 60.32%, 64.24%, 66.75%, and 60.51% for UAEWE, UAE, AE, and CHP, respectively (Table 1**)**, which were slightly lower than but followed the same order as the titrimetric values. The minor discrepancy between the two methods is acceptable, as titration measures the stoichiometric consumption of alkali by free carboxyl groups, while the FTIR method estimates DE based on the relative intensities of esterified and free carboxyl absorption bands. The consistent trend observed by both methods confirms that UAEWE-extracted pectin has the lowest esterification degree among the four samples, which is likely attributed to the synergistic effect of ultrasound cavitation and the acidic electrolyzed water environment promoting partial de-esterification of pectin molecules.

Compared with previous studies on hawthorn pectin, the DE values obtained in this study are generally higher than those reported by Uysal & Yildirim (2014), who reported DE values of 54.16% and 53.34% for red hawthorn pectins extracted using acidic solvents [43]. This difference may be attributed to variations in hawthorn cultivar, geographical origin, fruit maturity, and extraction conditions, all of which are known to influence the esterification degree of pectin. Although the absolute DE values differ, the classification of hawthorn pectin as a high-methoxyl pectin (DE > 50%) is consistent across studies.

The high DE values (63–71%) of the hawthorn pectin samples obtained in this study indicate that they are typical high-methoxyl pectins. High-methoxyl pectins are well known for their gelation properties, which require low pH (< 3.5) and high soluble solids content (> 55% sucrose) to form thermally reversible gels through hydrogen bonding and hydrophobic interactions. Therefore, these pectin samples hold promise as gelling agents in acidic food products such as jams, jellies, and fruit preserves. Furthermore, high-methoxyl pectins are suitable for the encapsulation and controlled release of bioactive compounds, as they can form calcium-resistant gels under gastric pH conditions, enabling targeted delivery to the intestine. Notably, UAEWE-extracted pectin, with the lowest DE (63.13%) and lowest molecular weight (223.92 kDa) among the four samples, exhibited the lowest apparent viscosity at 1% concentration, making it particularly attractive as a thickener or stabilizer in liquid food and beverage applications. This unique combination of properties may broaden the application scope of UAEWE-derived pectin in the food industry, particularly in functional beverage formulations and encapsulation systems for heat-sensitive bioactive compounds.

### 3.4 Molecular weight and particle size analysis of hawthorn pectin

The Mn values of UAEWE, UAE, AE, and CHP were 218.83 ± 3.46 kDa, 257.06 ± 3.87 kDa, 284.29 ± 1.98 kDa, and 222.16 ± 2.45 kDa, respectively, following the same trend as Mw (Table 1). This consistency further confirms that the extraction method significantly influences the molecular weight of hawthorn pectin, with UAEWE yielding the lowest molecular weight among all samples [44].

Analogous findings were achieved for the pectin extracted [45], [46]. The molecular weight (Mw) of UAE was markedly higher than that of UAEWE. This suggests that the extraction method using electrolytic water had a more substantial degradation impact compared to traditional acid extraction methods, as also observed in the studies by Lin et al. [47]. The molecular weight of sugar beet pectin has been reported to range from 100 to 700 kDa [48]. In comparison, the hawthorn pectin samples obtained in the present study exhibited relatively lower molecular weights. The polydispersity index (Mw/Mn) values of the four pectin samples were 1.02 ± 0.01, 1.02 ± 0.02, 1.02 ± 0.01, and 1.02 ± 0.03 for UAEWE, UAE, AE, and CHP, respectively. These values, all close to 1.0, indicate that all pectin samples possessed a relatively narrow molecular weight distribution. Similar findings have been reported in hawthorn pectin studies, where different extraction or fractionation methods affected molecular weight distribution [26], [49]. The low polydispersity observed in our study suggests that the pectin samples were relatively homogeneous in molecular size, which is beneficial for applications requiring consistent functional properties, such as gel formation and encapsulation of bioactive compounds.

Particle size analysis was an effective method capable of reflecting the dispersion state of substances in a solution. The particle size analysis results showed that the average particle sizes of UAEWE, UAE, AE and CHP were 3.564 μm, 4.250 μm, 5.673 μm and 4.673 μm respectively. These data further validated a strong positive correlation between the hydrodynamic diameter and the molecular weight (Mw) of pectin [50].

### 3.5 Monosaccharide composition

Table 1 showed the monosaccharide components of four types of hawthorn pectins. The main carbohydrate substances in hawthorn pectins were galactose, arabinose, mannose, rhamnose, and galacturonic acid. Although the monosaccharide composition remained consistent across the four types of hawthorn pectin, there were minor differences in their proportions [51]. It is noteworthy that glucose was the most abundant neutral sugar in all samples, with contents of 18.79%, 16.17%, 15.96%, and 13.42% in UAEWE, UAE, AE, and CHP, respectively. This glucose content was considerably higher than that of other neutral sugars such as galactose (2.04–3.04%), arabinose (2.12–2.39%), and rhamnose (2.16–3.16%). The relatively high glucose proportion may indicate the co-extraction of glucose-rich non-pectic polysaccharides, such as cellulose-derived components, starch, or β-glucans, which are naturally present in hawthorn cell walls. The acidic extraction conditions (pH 1.5) may partially hydrolyze these cellulosic or starch-like components, releasing glucose or glucose-containing oligosaccharides.

In addition, ultrasound-assisted extraction (UAEWE and UAE) may enhance cell wall disruption and mass transfer, thereby promoting the release of these non-pectic components. Despite the relatively high glucose content, all four pectin samples met the basic purity requirements for food-grade pectin, as their GalA contents exceeded 65% (w/w) [52]. This indicates that the extracts are predominantly composed of pectin and are suitable for food applications. However, for pharmaceutical or analytical applications where higher purity is required, additional purification steps (e.g., ethanol precipitation, ultrafiltration, or ion-exchange chromatography) would be necessary to remove neutral sugar impurities. It should also be noted that the presence of glucose-rich co-extractives does not compromise the comparative conclusions of this study, as all samples were obtained under comparable conditions and the UAEWE method consistently demonstrated superior performance in terms of yield, GalA content, and functional properties. The content of HG was roughly equivalent to the value obtained by subtracting the molar ratio of Rha from that of GalA [53]. The observed differences in HG and RG-I domain proportions among the four hawthorn pectin samples have important implications for their functional properties. The HG domain, consisting of linear GalA chains, is primarily responsible for pectin gelation through calcium-mediated crosslinking (for low-methoxyl pectins) or acid-sugar cooperative gelation (for high-methoxyl pectins). The consistently high HG contents (95.05–96.09%) across all samples indicate that they all possess good gel-forming potential. In particular, AE exhibited the highest Mw (290.91 kDa) and DE (70.58%) among the extracted samples, which, together with its high HG content, contributed to its highest apparent viscosity **(**Fig. 5**).**

**Fig. 2:**
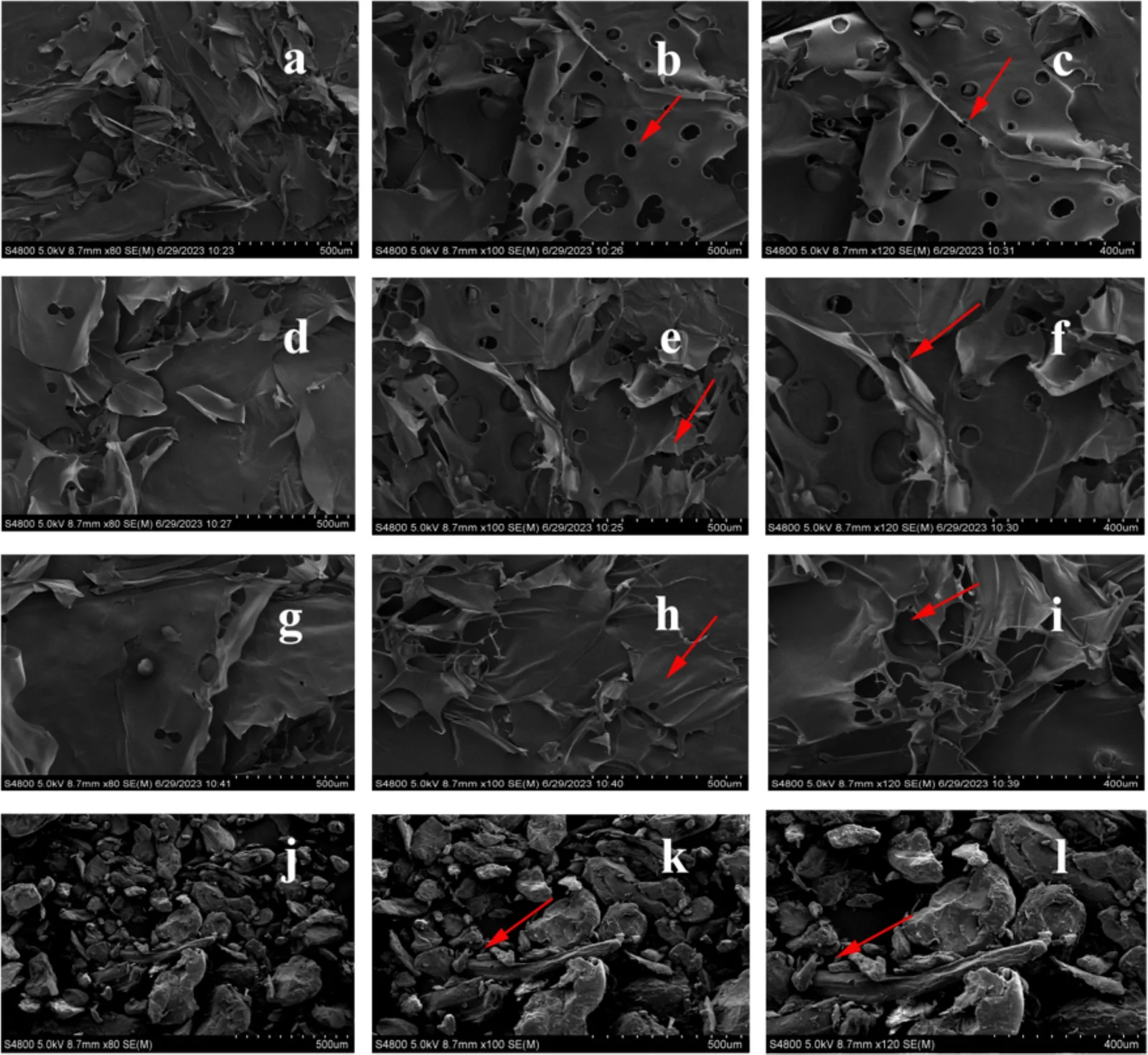
The SEM images obtained from three distinct extraction methods (a: UAEWE, 80×; b: UAEWE, 100×; c: UAEWE, 120×; d: UAE, 80×; e: UAE, 100×; f: UAE, 120×; g: AE, 80×; h: AE, 100×; i: AE, 120×; j: CHP, 80×; k: CHP, 100×; l: CHP, 120 × ). Arrows indicate the specific microstructural characteristics discussed in the text.

Conversely, UAEWE, with an HG content (95.30%) and lowest Mw (223.92 kDa), showed the lowest apparent viscosity, suggesting that UAEWE-derived pectin may be more suitable for applications requiring low viscosity and high solubility, such as beverage stabilizers or encapsulation wall materials. The molar ratio of Rha/GalA indicated the proportion of the RG domains [54]. The RG-I domain, characterized by rhamnose residues in the GalA backbone and bearing arabinose- and galactose-rich side chains, is known to influence the solubility, viscosity, emulsifying properties, and biological activities of pectin. The RG-I contents of the four types of hawthorn pectin were UAEWE (4.69 ± 0.03%), UAE (4.63 ± 0.01%), AE (4.94 ± 0.05%) and CHP (3.91 ± 0.05%) with significant differences, indicating that AE had the highest proportion of RG domains. The (Gal + Ara)/Rha ratio reflects the degree of side-chain branching [55]. The (Gal + Ara)/Rha values of UAEWE, UAE, AE and CHP were 1.68, 1.65, 1.72 and 1.92, respectively, suggesting that CHP had the greatest branching degree and UAE had the least branching degree. The higher branching degree of CHP may explain its complex rheological behavior and distinct granular microstructure observed by SEM **(**Fig. 2**j–l),** as highly branched pectins tend to exhibit different intermolecular interactions in solution. Furthermore, the moderate branching degree of UAEWE, combined with its lower molecular weight, may contribute to its superior antioxidant activity **(**Fig. 6**)**, as the exposed side-chain residues and reducing ends may facilitate radical scavenging through hydrogen donation. These structure–function relationships demonstrate that the extraction method significantly influences not only the structural features but also the potential applications of hawthorn pectin [56].

### 3.6 Scanning electron microscopy

Scanning electron microscopy (SEM) was a key technique for in-depth exploration of the microstructure of hawthorn pectin [57]. As shown in Fig. 2, the microstructure of hawthorn pectin showed significant differences after being treated with different extraction methods. The AE sample presented a complete structure, featuring thick edges and a comparatively smooth surface. In contrast, the layered structure of UAE and UAEWE was distinct, with thin edges and surfaces filled with empty small bubbles, which might be associated with the cavitation effect of ultrasound. Of particular note, UAEWE exhibited more significant fragmentation and pore structure, which increased the contact area with the solvent and thus improved the yield [58]. The microstructural differences observed among the four hawthorn pectin samples are consistent with previous reports on pectins extracted from hawthorn and other plant sources. Similar changes in microstructure caused by different extraction methods have also been reported for hawthorn pectin. For instance, acid-extracted hawthorn pectin typically exhibited relatively smooth and intact surfaces, whereas ultrasound-assisted extraction resulted in more loosely arranged and fragmented microstructures with porous features. Comparable phenomena have also been observed in apple pomace pectin, citrus peel pectin, and sugar beet pectin, where ultrasound-induced cavitation disrupted the plant cell wall matrix and enhanced the release of pectin. These effects are generally attributed to the physical disruption of the cell wall network by cavitational forces, which promotes mass transfer and solubilization of pectin from the cell wall matrix.

Notably, UAEWE-derived pectin exhibited the most pronounced fragmented and porous microstructure, characterized by thin edges and numerous hollow cavities distributed throughout the surface. This highly porous architecture directly contributed to its highest extraction yield (7.21%), as the increased specific surface area facilitated solvent penetration and mass transfer, thereby promoting the dissolution and release of pectin from the plant cell wall matrix [59]. In contrast, AE-derived pectin, which showed a relatively complete and smooth surface with thick edges, exhibited the lowest extraction yield (4.53%), likely due to the limited solvent accessibility and mass transfer efficiency. The microstructural features were also consistent with the molecular weight and particle size results. UAEWE-derived pectin, with the most fragmented structure, had the lowest molecular weight (223.92 kDa) and smallest particle size (3.56 μm), suggesting that the combination of ultrasound cavitation and acidic electrolyzed water promoted structural disruption and partial depolymerization of pectin chains. Conversely, AE-derived pectin, with the most intact structure, exhibited the highest molecular weight (290.91 kDa) and largest particle size (5.67 μm). These correlations indicate that extraction-induced microstructural changes are intimately linked to the physicochemical properties of the resulting pectin. Furthermore, the porous and fragmented structure of UAEWE-derived pectin may also contribute to its enhanced antioxidant activity (Fig. 6), as the disrupted structure likely exposes more reducing ends and functional groups, facilitating radical scavenging.

The microstructure of commercially-available hawthorn pectin exhibits a distinctly different morphology compared with that of the other three types of hawthorn pectin. The CHP sample shows a granular and irregular structure, with particles of varying sizes. Unlike the relatively flat sheet-like structures with bubble features in UAE and UAEWE, or the complete and smooth-surfaced structure of AE, the granular nature of CHP may indicate different aggregation states or interaction patterns during the extraction or processing. These microstructural differences may partly explain the differences in physicochemical properties and antioxidant activity among the four hawthorn pectin samples, although antioxidant activity is also influenced by molecular weight, GalA content, esterification degree, and other structural factors [26], [60]. These findings suggest that the microstructural characteristics of UAEWE-derived hawthorn pectin, together with its favorable physicochemical properties, make it a promising candidate for food applications, particularly as a functional additive with rapid dissolution properties or as a wall material for bioactive compound encapsulation.

### 3.7 FTIR analysis

**Fig. 3:**
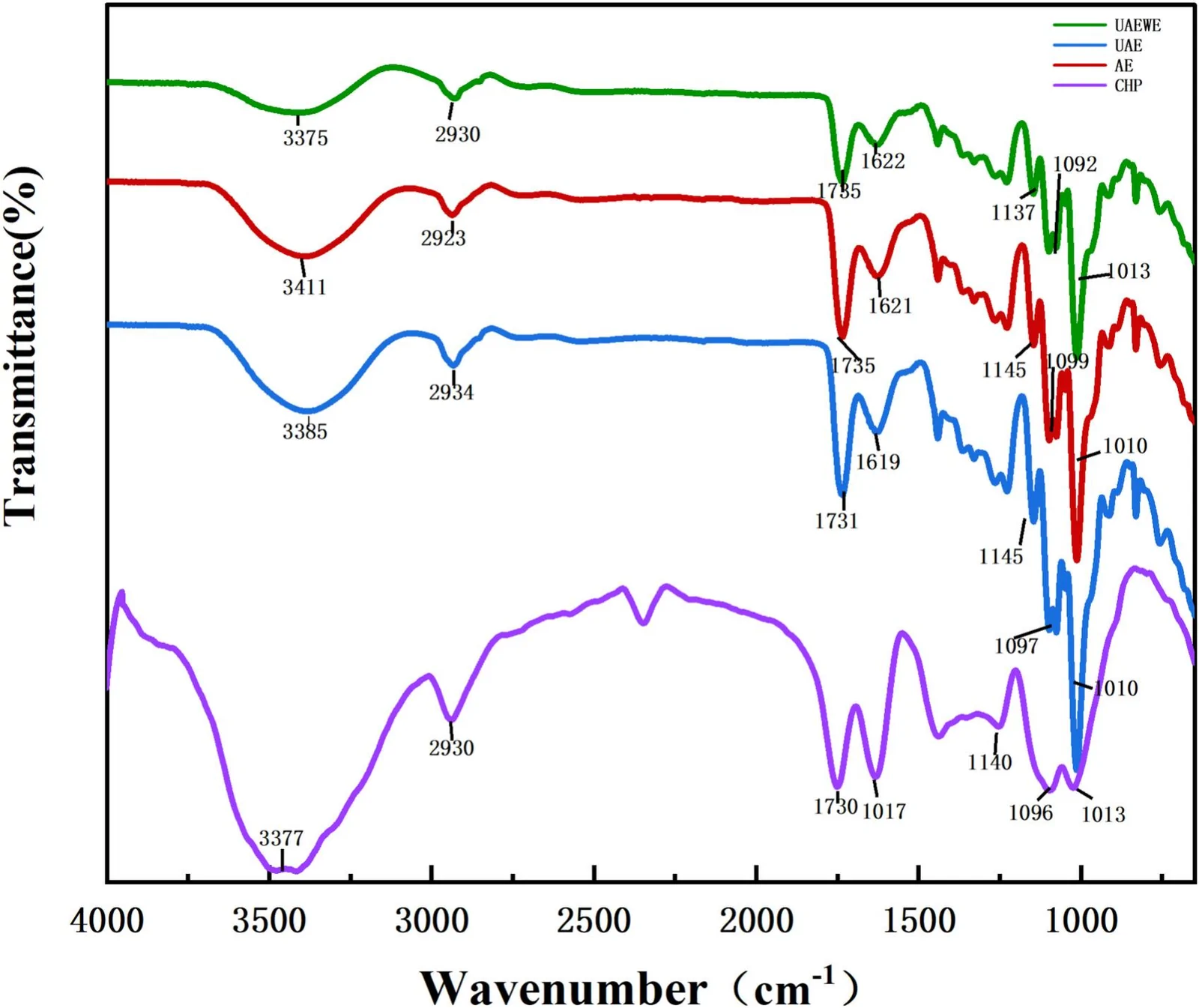
The FTIR spectra of UAEWE, UAE, AE and CHP.

Fig. 3 shows the infrared scanning spectra of four kinds of pectin. It can be seen that they present the same characteristic peaks, but the peak intensities are different. The wide and intense energy band detected at around 3385 cm^–1^ was associated with the stretching vibration of the hydrogen bond of the hydroxyl group associated with galacturonic acid. The stretching vibration absorption peak of the hydroxyl O-H bond at around 3385 cm^−1^ in CHP was stronger than that in UAEWE, UAE, AE, indicating that CHP contained more hydroxyl groups. The absorption peak located at around 2930 cm^–1^ corresponded to the stretching environment of C-H bonds within functional groups such as –CH, –CH_2_, and –CH_3_ [61]. The asymmetric tension of methylated carboxyl group (O-CH_3_) exhibited a stronger absorption signal at around 1737 cm^−1^ compared to the stretching vibration of free carboxyl group (COO–) at around 1623 cm^−1^, which was a characteristic of high methylated pectin (DM > 50%) [62]. This assignment was consistent with previous reports on hawthorn pectin and other plant-derived pectins, in which the absorption band near 1740 cm^–1^ was attributed to esterified carboxyl groups and the band around 1600–1630 cm^–1^ was assigned to free carboxyl groups [1], [26]. The energy band within the 1200–1400 cm^–1^ range was associated with the bending of CH_2_, the bending of OH, and the stretching of –CH_3_CO. The band spanning from around 1200 cm^–1^ to around 800 cm^–1^ was regarded as the “fingerprint” area of carbohydrates, which mirrored certain alterations in the monosaccharide composition [63]. The band in the range of around 1010 cm^–1^ to around 1150 cm^–1^ suggested that the pectin sample had pyranose present [64]. The peaks at around 1102 cm^–1^ and around 1019 cm^–1^ were caused by the asymmetric stretching of the C-O-C bond [65]. As is evident from the figure, the peak areas of the three types of pectin at around 1737 cm^–1^ and around 1623 cm^–1^ exhibit minor differences. Specifically, UAEWE-derived pectin showed a relatively weaker absorption at 1737 cm^–1^ and a relatively stronger absorption at 1623 cm^–1^ compared with AE and UAE. This spectral feature is consistent with its lower DE value (63.13%), as determined by titration, and suggests that UAEWE extraction promoted partial de-esterification of pectin molecules. This effect can be attributed to the hydrolysis of methyl ester bonds under the acidic conditions of electrolyzed water (pH ∼ 1.5), which was further enhanced by ultrasound cavitation. In contrast, AE-derived pectin exhibited the strongest absorption at 1737 cm^–1^ and the weakest at 1623 cm^–1^, consistent with its highest DE value (70.58%). CHP showed intermediate spectral characteristics, which aligned with its intermediate DE value (65.58%). The DE values calculated from the FTIR peak area ratio (A_1740_/(A_1740_ + A_1630_)) were 60.32%, 64.24%, 66.75%, and 60.51% for UAEWE, UAE, AE, and CHP, respectively. These values are slightly lower than but follow the same trend as those determined by the titrimetric method **(**Table 1**)**, confirming that UAEWE-derived pectin has the lowest esterification degree among the four samples. Because the relative intensities of the esterified and free carboxyl absorption bands are closely related to the esterification degree of pectin, this spectral difference was consistent with the lower DE value of UAEWE and suggested that ultrasound-assisted electrolyzed water extraction may promote partial de-esterification of hawthorn pectin [21], [26], [66]. This finding is in accordance with the data presented in Table 1. In addition, no obvious disappearance of the major characteristic peaks was observed among the four samples, indicating that different extraction methods mainly affected the relative abundance of functional groups rather than completely altering the basic pectin backbone [21], [66]. These findings indicate that UAEWE is a mild yet effective extraction method that preserves the pectin backbone while selectively modifying the esterification degree, thereby enhancing its functional properties such as antioxidant activity and solubility.

### 3.8 NMR analysis

**Fig. 4:**
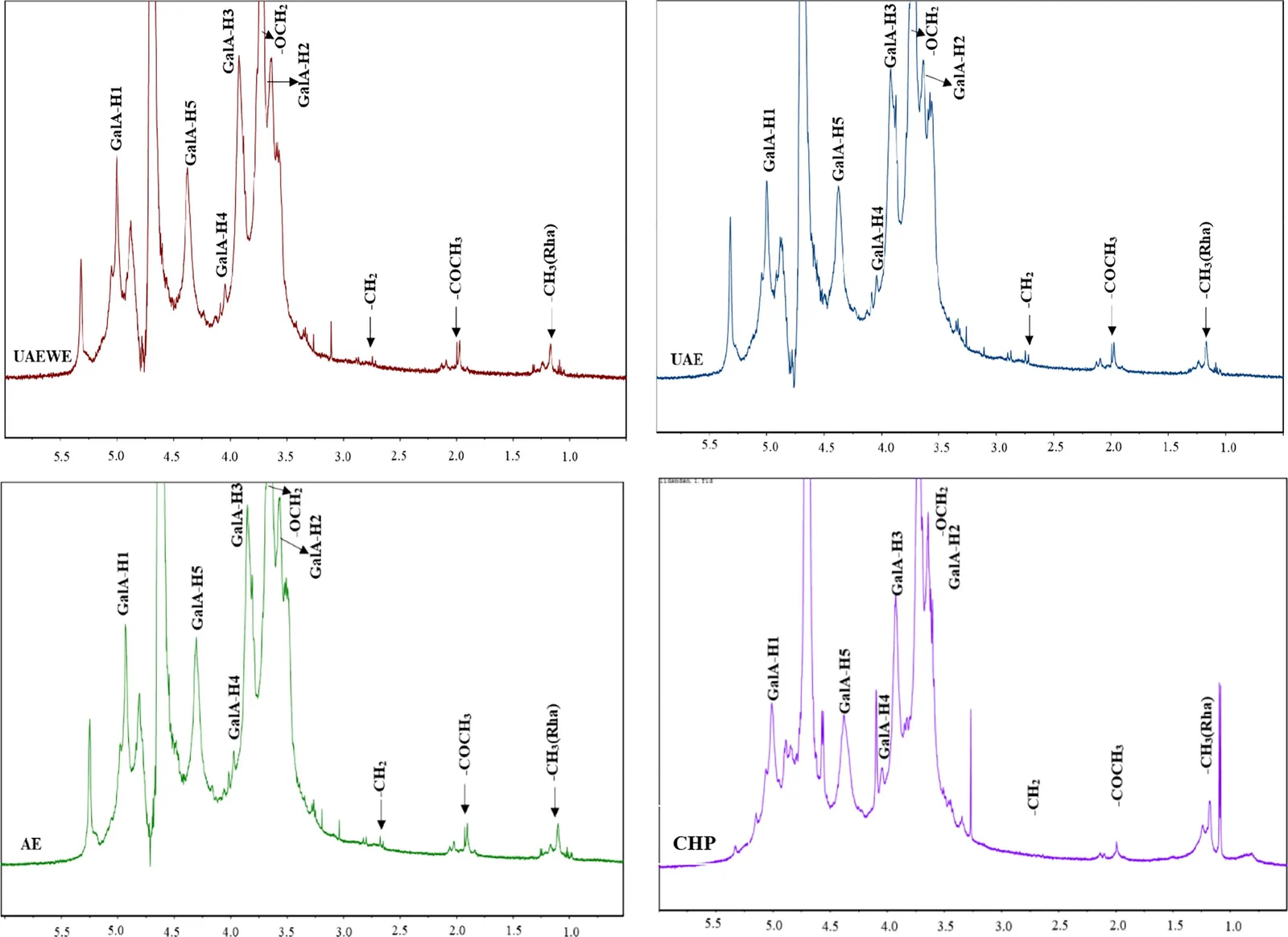
^1^H NMR spectra of UAEWE, UAE, AE and CHP.

Fig. 4 clearly demonstrates that the ^1^H NMR spectra of the hawthorn pectin samples obtained through these three extraction techniques are nearly identical and exhibit no significant differences. The similarity of the main resonance signals indicated that the primary pectin backbone was largely retained after UAEWE, UAE, and AE treatments, which is consistent with previous reports showing that different processing or extraction methods may modify pectin properties without completely altering the main structural framework [27], [49]. This finding is consistent with previous reports on hawthorn pectin and other plant-derived pectins, where different processing or extraction methods were shown to modify pectin properties without completely altering the main structural framework. For instance, previous studies on citrus and apple pectins have demonstrated that ultrasound-assisted extraction may reduce molecular weight and alter DE values, but the fundamental galacturonic acid backbone remains intact, as confirmed by ^1^H NMR analysis. But it can be seen from the figure that ^1^H NMR of CHP is relatively more complex than AE and UAE, indicating that CHP has a more complex branched-chain structure. The relatively higher complexity of the CHP spectrum may be related to differences in raw material source, purification process, or commercial processing conditions. Commercial hawthorn pectin preparations often undergo additional processing steps, such as blending, fractionation, or standardization, which can influence the relative abundance of neutral sugar side chains and the aggregation behavior of pectin molecules. This observation is consistent with previous reports indicating that commercial pectins may exhibit more complex NMR profiles due to their heterogeneous origin and processing history. This difference may be related to the different raw material source, purification process, or commercial processing conditions of CHP, which could influence the relative abundance of neutral sugar side chains and aggregation behavior of pectin [49], [67]. The assignment of these characteristic signals is in good agreement with previous studies on hawthorn and citrus pectins. Specifically, the signal at 1.17 ppm assigned to the methyl group of L-rhamnose connected to O-2,4 and O-2 has been consistently reported in hawthorn pectin research [68]. The signals at 2.0 ppm and 2.64 ppm attributed to acetyl and methylene groups esterified with 2-O- and 3-O-GalA are consistent with citrus pectin studies, confirming the presence of acetyl substitutions on the galacturonic acid backbone [54]. Furthermore, the strong signal at 3.81 ppm corresponding to the methoxy group of GalA carboxyl, which proves the presence of a galacturonic acid backbone, has been widely observed in pectins from various plant sources [69]. The presence of methyl-ester-related signals was also consistent with the high DE values shown in Table 1, whereas the relatively weaker esterification-related signals in UAEWE may be associated with its lower DE value. Similar relationships between NMR signals of methyl-ester groups and pectin esterification characteristics have been reported in plant-derived pectins [67], [70]. From FTIR and ^1^H NMR, hawthorn pectin's main chemical structure stays the same with different extraction methods. However, ^1^H NMR analysis showed that CHP had abundant branching. The relationship between methyl-ester-related NMR signals and pectin esterification characteristics has been well established in plant-derived pectins. Although the main signals were similar among the four samples, notable differences were observed. UAEWE showed a relatively weaker methoxy signal at *δ* 3.81 ppm compared with AE and UAE, which was consistent with its lower DE value (63.13%) determined by titration. This observation suggests that the combination of ultrasound cavitation and acidic electrolyzed water promoted partial de-esterification of pectin molecules through the hydrolysis of methyl ester bonds, in agreement with the FTIR results (Fig. 3). In contrast, AE exhibited the strongest methoxy signal, consistent with its highest DE value (70.58%). CHP showed a more complex spectrum with additional signals in the *δ* 3.5–4.5 ppm region, indicating a higher abundance of neutral sugar side chains, which was consistent with its higher (Gal + Ara)/Rha ratio (1.92) shown in Table 1. Moreover, no obvious disappearance of the major characteristic NMR signals was observed among the extracted pectin samples, indicating that the different extraction methods mainly affected signal intensity and side-chain-related structural features rather than completely destroying the main pectin backbone. For UAEWE, the combined effects of ultrasound cavitation and the acidic electrolyzed water environment may have promoted partial de-esterification and molecular loosening while retaining the primary pectin structure. These structural modifications are consistent with the lower DE, lower Mw, higher extraction yield, and enhanced antioxidant activity observed for UAEWE-derived pectin [27], [70]. These results, together with the FTIR data, confirm that UAEWE is a mild but effective extraction method that preserves the pectin backbone while promoting partial de-esterification, thereby modulating the functional properties of the resulting pectin.

**Fig. 5:**
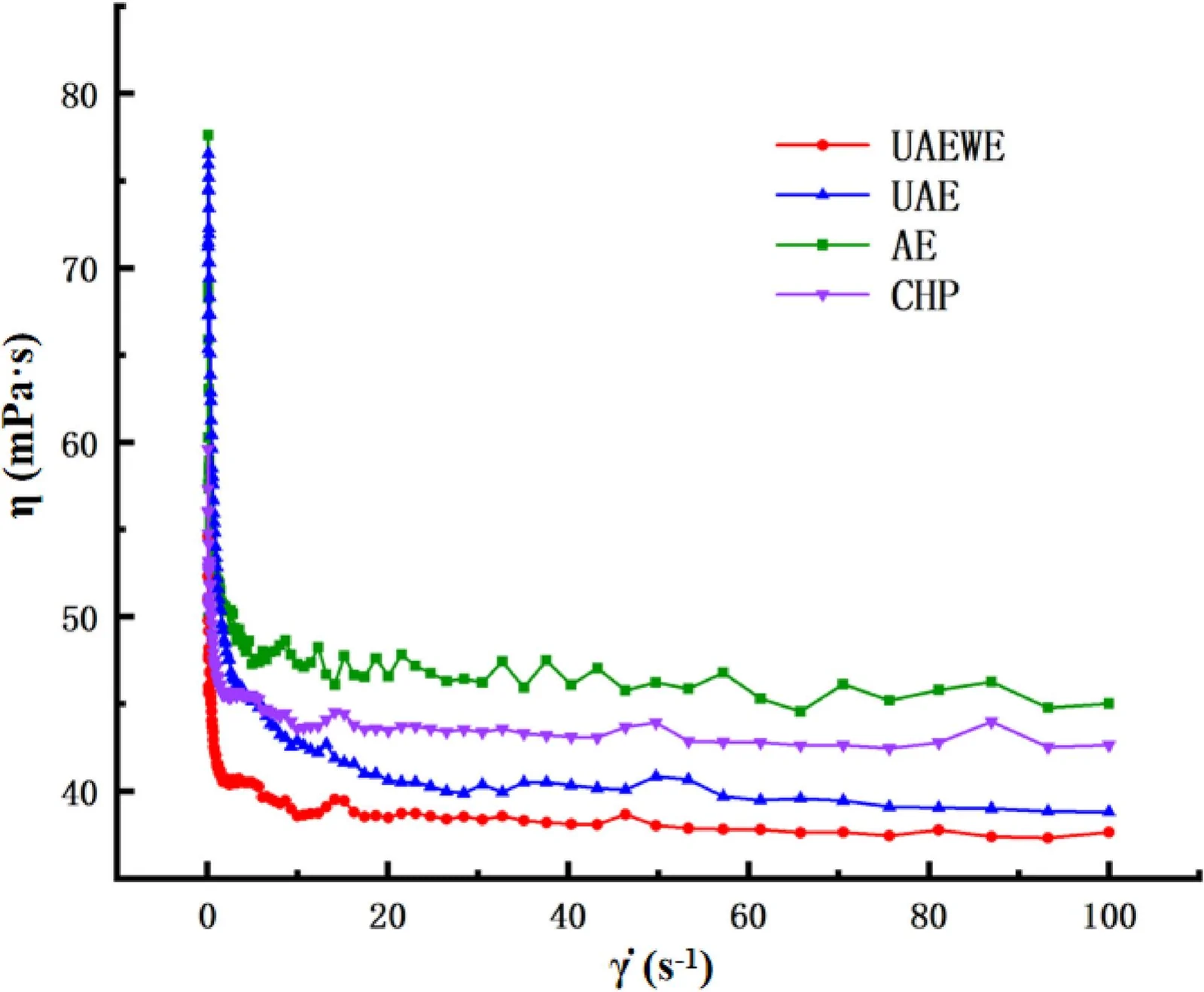
Steady flow behavior of UAEWE, UAE, AE and CHP solutions.

### 3.9 Rheological analysis

Typically, the application of diverse extraction methods might lead to substantial disparities in the physicochemical characteristics of hawthorn pectin. The power law equation, as one of the most fundamental models, could well describe the flow behavior of most materials under shear action (Table 2). These materials were typically classified as shear-thinning fluids, meaning that their flow characteristics could be accurately represented by power law equations within a certain range of shear rates.

**Table 2:** Power-law model parameters for the rheological behavior of UAEWE, UAE, AE, and CHP.

	K (Pa·s^n^)	n	R^2^
UAEWE	0.38 ± 0.04^d^	0.52 ± 0.03^d^	0.9993
UAE	0.42 ± 0.01^c^	0.68 ± 0.02^b^	0.9995
AE	0.63 ± 0.02^a^	0.72 ± 0.05^a^	0.9947
CHP	0.45 ± 0.03^b^	0.59 ± 0.04^c^	0.9992

K, consistency index.

n, flow behavior index.

Different lowercase letters indicate significant differences at *p* < 0.05.

At a concentration of 1% and pH 3, the four types of pectins exhibited characteristics similar to Newtonian fluids, characterized by smaller viscosity coefficients and rheological properties index close to 1. With the rise of the shear rate, the apparent viscosity of these pectins declined gradually, showing distinct shear-thinning features. It was particularly noteworthy that at lower shear rates, the apparent viscosities of the four types of hawthorn pectin varied significantly. Specifically, among them, the apparent viscosity followed the order of AE > CHP > UAE > UAEWE. Moreover, the differences in the apparent viscosities of the four types of hawthorn pectins are consistent with the molecular weights shown in Table 1. This was because as the shear rate increased, the internal network cross-linking structure of pectin molecules was disrupted, leading to a decrease in their apparent viscosity. Furthermore, the rheological properties of pectin were strongly associated with its molecular weight, and the larger the molecular weight, the more obvious its rheological properties were [71].

It was worth mentioning that UAEWE, as a special type of pectin, had the characteristics of low apparent viscosity and low DE value, which gave it great potential for application in beverage thickeners. In the food industry, high requirements were placed on the shear thinning performance of certain polymers and adhesives [72], as this characteristic not only facilitated the pumping of liquid food but also brought a pleasant taste to the food.

### 3.10 Analysis of the antioxidant activity of hawthorn pectin

**Fig. 6:**
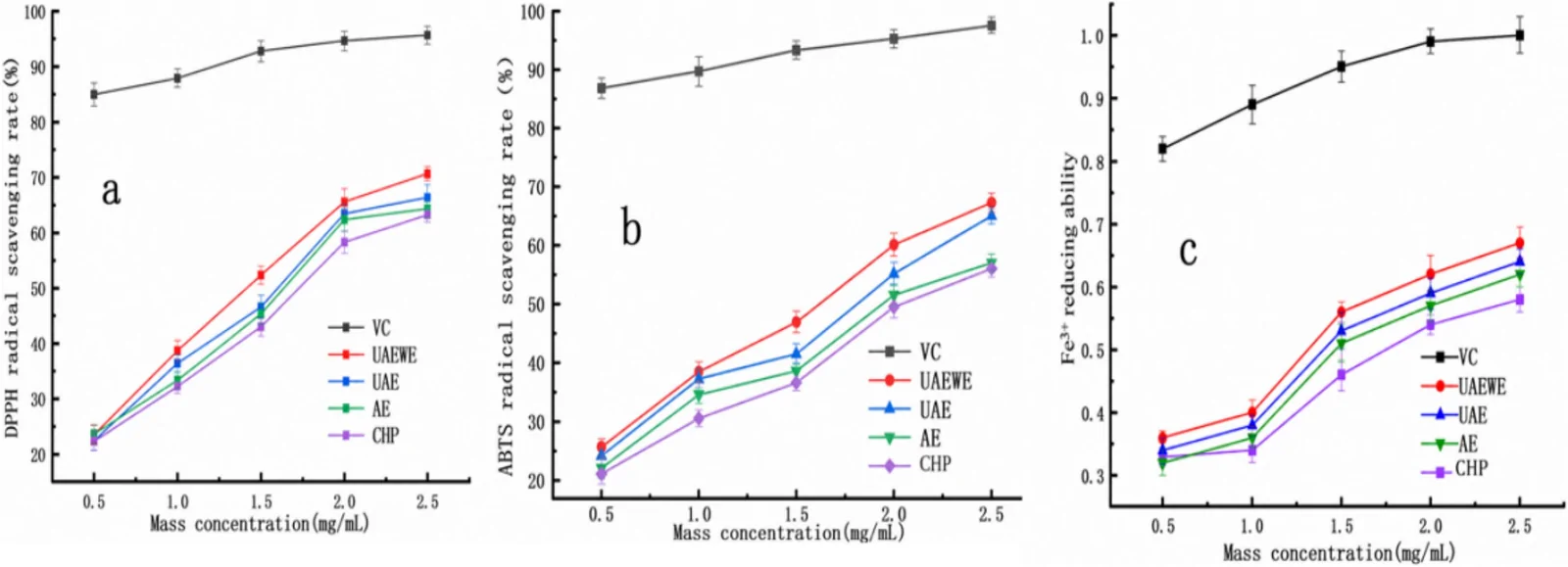
Comparison of DPPH radical scavenging rate (a), ABTS cationic radical scavenging rate (b), and Fe^3+^-reducing power (c) of UAEWE, UAE, AE, and CHP samples.

DPPH is a stable free radical widely used as an effective means to evaluate the antioxidant's ability to scavenge free radicals. As depicted in Fig. 6**a**, within the concentration range of 0.5–2.5 mg/mL, the DPPH free-radical scavenging rate of hawthorn pectin demonstrated a dose-dependent manner. When the mass concentration exceeded 1.0 mg/mL, the DPPH radical scavenging activity of UAEWE was notably higher than that of the other three types at every mass concentration level (*p* < 0.05). At the same mass concentration, the AE clearance rate was still significantly lower than the other three types (*p* < 0.05). At a mass concentration of 2.5 mg/mL, the free radical scavenging rate of UAEWE attained 70.69%. Compared with UAE, UAEWE, AE, and CHP significant differences exist between the VC control and the other four types of pectin. Overall, UAEWE had the strongest DPPH radical scavenging ability, followed by UAE, AE and CHP.

The ABTS cation free radical scavenging rate is shown in Fig. 6**b.** The clearance rate of hawthorn pectin on ABTS cationic free radicals obtained by four methods increased with the increase of mass concentration. When the mass concentration was below 1 mg/mL, the reduction ability of UAE and UAEWE was similar; When the mass concentration was higher than 1 mg/mL, the UAEWE radical scavenging activity was significantly increased; At 2.5 mg/mL, theradical scavenging rate of UAEWE reached 67.32%, compared with UAE, AE, and CHP. Significant differences existed between the VC control and the other four types of pectin, indicating that UAEWE had the strongest ABTS cationic free radical scavenging ability, followed by UAE, AE and CHP.

The results of Fe^3+^reduction ability are shown in Fig. 6**c**. The hawthorn pectin obtained through the four methods all had a certain Fe^3+^reduction ability. When the mass concentration exceeded 1.5 mg/mL, the trend of increasing reducing ability with increasing mass concentration gradually slowed down, and the reducing ability reached its maximum value of 0.67 at 2.5 mg/mL. There were significant differences between the VC control group and the four types of pectin. Overall, at the same mass concentration, the reducing ability of Fe^3+^ was UAEWE > UAE > AE > CHP. Due to the crosslinking between carboxyl groups and ions in uronic acid [73], pectin exhibited the ability to reduce metal ions. The pectin extracted by UAEWE had a lower molecular weight structure, resulting in more exposed reducing ends and greater reducing ability.

Compared with previous studies, the content of uronic acid and glucose in UAEWE was higher than that in AE, UAE, and CHP, according to the analysis of monosaccharide composition and chemical composition results, indicating that acidic electrolyzed water had an oxidation–reduction potential [74]. The extraction process could protect the carbonyl and aldehyde groups on the polysaccharide chains, while acidic water extraction might disrupt the structure of pectin and affect its biological activity. Therefore, this might be the reason why UAEWE had a higher clearance rate. The antioxidant activities of hawthorn pectin samples obtained in this study were compared with those reported in previous studies. Lv et al. (2024) reported that hawthorn pectin extracted by ultra-high pressure-assisted acid extraction exhibited a DPPH radical scavenging activity of 93.70% at 4 mg/mL [26], while Ye et al. (2025) reported a DPPH scavenging rate of 92.72% for hawthorn pectin obtained by ultrahigh-pressure-assisted enzymatic extraction [75]. Although the DPPH scavenging activity of UAEWE-derived pectin in this study (70.69% at 2.5 mg/mL) was lower than those reported at higher concentrations, UAEWE still showed stronger antioxidant activity than UAE, AE, and CHP under the same experimental conditions. The differences in absolute values between our study and literature reports may be attributed to several factors. First, the test concentrations differed (4 mg/mL in literature vs. 2.5 mg/mL in this study), and antioxidant activity is typically concentration-dependent. Second, the extraction methods differed—ultra-high pressure-assisted extraction may preserve bioactive structural features more effectively than conventional or ultrasound-assisted extraction methods. Third, raw material variability (cultivar, origin, maturity, and processing) could influence the molecular structure and bioactivity of pectin. Fourth, commercial pectin (CHP) exhibited the lowest antioxidant activity, which may be related to the loss of bioactive components during commercial processing or the use of raw materials with inherently low antioxidant activity. Importantly, the relatively higher antioxidant activity of UAEWE-derived pectin is consistent with its structural characteristics, including the highest GalA content (87.35 g/100 g), lowest molecular weight (223.92 kDa), and lower esterification degree (63.13%). These features may expose more reducing ends and active functional groups, facilitating electron donation and radical scavenging. This structure–activity relationship further validates the superiority of the UAEWE method for producing hawthorn pectin with enhanced functional properties [12].

## 4 Conclusions

In this study, ultrasound-assisted electrolyzed water extraction (UAEWE) was developed as a green and efficient strategy for recovering pectin from hawthorn, and its performance was systematically compared with UAE, AE, and CHP. UAEWE achieved the highest pectin yield (7.21%) and GalA content (87.35 g/100 g), while producing pectin with lower molecular weight (223.92 kDa), smaller particle size (3.56 μm), and a more porous microstructure. Notably, UAEWE-derived pectin exhibited the lowest degree of esterification (63.13%) and apparent viscosity, together with enhanced DPPH (70.69%) and ABTS (67.32%) radical scavenging activities at 2.5 mg/mL. FTIR and NMR analyses confirmed that the primary pectin backbone was well preserved. These favorable attributes make UAEWE-derived hawthorn pectin a promising candidate for applications in food additive delivery, bioactive compound encapsulation, and functional food formulations. Overall, UAEWE represents a sustainable, cost-effective, and environmentally friendly alternative to conventional acid extraction for hawthorn pectin production.

## 5 Author Statement

We declare that this manuscript is original, has not been published before and is notcurrently being considered for publication elsewhere.

We confirm that the manuscript has been read and approved by all named authors andthat there are no other persons who satisfied the criteria for authorship but are not listed.We further confirm that the order of authors listed in the manuscript has been approvedby all of us.

We understand that the Corresponding Author is the sole contact for the Editorialprocess. She is responsible for communicating with the other authors about progress, submissions of revisions, and final approval of proofs.

**Data availability**

Data will be made available upon reasonable request.

## CRediT authorship contribution statement

**Zishuai Liu:** Writing – original draft, Methodology, Investigation, Formal analysis, Data curation. **Jiamin Zhang:** Validation, Data curation. **Congxuan Fei:** Investigation. **Dandan Li:** Writing – review & editing, Conceptualization. **Hong Zhu:** Investigation. **Jie Zhou:** Investigation. **Guangxian Wang:** Resources. **Yafei Zheng:** Resources. **Xinming Liu:** Investigation. **Jianhua Xiu:** Supervision.

## Declaration of competing interest

We state that there are no financial or any other forms of conflicts of interest regarding our work. The research presented is entirely original, and the data included in this manuscript has not been previously published in any other venue.

## Appendix A Supplementary data

Supplementary Data 1 Full-wavelength UV spectra of hawthorn pectins extracted by UAEWE, UAE, and AE.
