# Supplementary material for: Study on the physicochemical properties and antioxidant activity of hawthorn pectin extracted by ultrasound-assisted electrolysis of water
Source: Ultrason Sonochem. 2026 Aug 19;133:108012. doi: 10.1016/j.ultsonch.2026.108012 (PMC13524504; doi:10.1016/j.ultsonch.2026.108012)
Supplement: Supplementary file 1 [file mmc1.docx]

**Fig.S1.** Full-wavelength UV spectra of hawthorn pectins extracted by UAEWE, UAE, and AE
